# Hydrogen saline water accelerates fracture healing by suppressing autophagy in ovariectomized rats

**DOI:** 10.3389/fendo.2022.962303

**Published:** 2022-09-02

**Authors:** Jialiang Guo, Siyu Tian, Zhongzheng Wang, Yuchuan Wang, Xiaojuan Zhang, Yingze Zhang, Zhiyong Hou, Weichong Dong

**Affiliations:** ^1^ Department of Orthopaedics, The Third Hospital of Hebei Medical University, Shijiazhuang, China; ^2^ Chinese Academy of Engineering, Beijing, China; ^3^ National Health Commission (NHC) Key Laboratory of Intelligent Orthopeadic Equipment, The Third Hospital of Hebei Medical University, Shi Jiazhuang, Hebei, China; ^4^ Department of Pharmacy, The Second Hospital of Hebei Medical University, Shijiazhuang, China

**Keywords:** oxidative stress, menopausal osteoporosis, osteoporotic fracture, redox markers, hydrogen water

## Abstract

**Objective:**

The treatment of osteoporotic fractures is difficult, and to minimize the negative result or poor functional rehabilitation, this study focuses on hydrogen water (HRW) to test its effect on the process of menopausal osteoporotic fracture healing and its relationship with autophagy and to try to reveal the potential mechanism of action of HRW on osteoporotic fractures.

**Materials and methods:**

A rat osteoporotic fracture model was established, and HRW was systematically applied with or without 3MA. The results were analyzed with X-rays, micro-CT scans, serum biomarker analysis, biomechanical tests, histopathology, immunohistochemistry, and Western blotting. The sham, OVX, OH (OVX+HRW) and OHA (OVX+HRW+3MA) groups were formed and compared.

**Results:**

Increased oxidative stress and autophagy levels were necessary physiological responses in the process of fracture healing. It was found that systemic HRW treatment slightly suppressed autophagy and then activated the Keap1-Nrf2 signaling pathway by maintaining the Keap1-Nrf2-P62 interaction and improved the osteoporotic fracture healing process.

**Conclusion:**

HRW treatment activated the Keap1-Nrf2 signaling pathway to antagonize cellular stress by suppressing autophagy levels, especially at the early stage of the fracture healing process, and this was beneficial to osteoporotic fracture healing in rats.

## Introduction

Osteoporosis (OP) is a common disease that is characterized by the loss of bone mass and the deterioration of bone microstructure. Patients with OP are more prone to fracture, and nonunion of osteoporotic fracture is a commonly seen complication and may result in a heavy burden for the patient’s family. The treatment of an osteoporotic fracture is difficult, and to minimize its negative effects or poor functional rehabilitation, it is essential to take active prevention measures as early as possible. In addition to the anti-osteoporosis drugs and autogenous bone grafts widely used in the clinic, other natural molecules are reported to be potentially applicable in animal models.

Molecular hydrogen is small, nontoxic, electrically neutral, and nonpolar and can diffuse freely across all cell membranes, such as the blood–brain barrier and placental barriers. Molecular hydrogen has been comprehensively studied in the past 10 years, and it has attracted great interest due to its antioxidative, antiapoptotic, and anti-inflammatory properties. Hydrogen has an anti-inflammatory effect and selectively reacts with hydroxyl radicals to effectively target intracellular inflammatory factors. Since the excellent therapeutic effect of hydrogen in a rat model of cerebral infarction was first demonstrated, increasing evidence has shown that many diseases are improved with hydrogen treatment (1). Furthermore, in our previous research, it was found that hydrogen water (HRW) can preserve bone volume and decrease fracture risks in streptozotocin-induced diabetic rats with bone structure changes due to increased levels of oxidative stress (2). Malondialdehyde (MDA), which reflects the level of oxidative stress, was decreased, but the specific mechanisms underlying the therapeutic effects of hydrogen need to be further explored under high oxidative stress conditions.

Autophagy is a self-cannibalization process involving the engulfment of cytoplasmic material and intracellular organelles in a double-membrane autophagosome. In recent years, the relationship between oxidative stress and autophagy has been recognized, and it is widely believed that ROS can induce autophagy, which in turn reduces the damage caused by oxidative stress to the body. Recently, there have also been an increasing number of studies on the relationship between hydrogen and autophagy. However, the role of hydrogen in autophagy remains controversial despite its known effect in decreasing oxidative stress levels. Some studies have shown that HRW downregulates autophagy (3, 4), but other studies have demonstrated that hydrogen induces autophagy (5, 6). Furthermore, although menopausal OP is more commonly observed than diabetic OP, no research about the mechanism of action of HRW on menopausal OP has been conducted. The aim of this study was to investigate the effect of HRW on menopausal osteoporotic fracture healing and its regulation of autophagy and to reveal the potential mechanism of action of the effect of HRW on osteoporotic fractures.

## Materials and methods

### Animal model and treatment

This study was approved by the Ethics Board of our Hospital and was conducted in accordance with the animal experiment center guidelines (S2020-024-1). Female aging Sprague–Dawley (SD) rats obtained from the animal center were kept in a stable environment and given free access to tap water and rodent chow diet. Changes in appearance were monitored every day and addressed immediately. A total of 96 12-week-old female SD rats (300 g ± 13 g) (24 animals in each group to ensure that the sample size of each experiment reached 6) were chosen and used in our study for biomechanical and histopathological experiments.

After a one-week acclimation period, estrogen withdrawal *via* ovariectomy (OVX) was performed *via* a dorsal approach (one incision located at the middle back) in 72 randomly chosen SD rats, and the other 24 female rats underwent sham surgeries with bilateral ovaries simply exposed. The OVX rats were randomly divided into 3 different intervention groups (n = 24). At 12 weeks after OVX operation, the rats were all then operated on to form a femoral fracture model through a middle lateral femoral fusion approach within 2 days. In addition, the rats without OVX were normally raised (sham). All rats received intragastric administration of vehicle (V = normal saline) in the control group (OVX) or intragastric administration of HRW at 5 ml/d in the OVX+HRW group (OH) (2). The OVX+HRW+3MA (OHA) group was intragastrically administered HRW at a dose of approximately 5 ml/d, and 3MA (50 mg/kg, HY-19312, MedChemExpress, China) was also subcutaneously injected for three days after the femoral fracture model was established.

### Surgical procedure

HRW (H2 concentration > 1.6 ppm) was provided by Beijing Hydrovita Beverage Co., Ltd. (Beijing, China) and stored under atmospheric pressure at 23 ± 2°C in an aluminum container with no dead volume. The rats were anesthetized through intraperitoneal injections of sodium pentobarbital (1%, 0.4 ml/100 g, Sigma–Aldrich, St Louis, MO, USA). The rats were fixed with the fur on the right leg shaved and sterilized. A 1.0-cm middle lateral incision was used to expose the middle femoral shaft. Then, a wire saw was used to conduct the femoral osteotomy. After the reduction osteotomy, a sterilized Kirschner wire (ø1 mm, Jinhuan Medical Products, Shanghai, China) was used to fix the proximal femoral canal and the distal femur. It was ensured that the distal wire was buried under the surface of the knee joint. The rats received intragastric administration of vehicle (V = normal saline, 0.9% normal saline water, the Fourth Pharmaceutical Factory, Shi Jiazhuang, China) in the sham and OVX groups and 5 ml/d HRW in the OH group, as mentioned above. The rats were given prophylactic antibiotics (cefazolin sodium; 1 mg, Taiyang Pharmaceutical, Beijing, China) for 3 days soon after surgery. The rats were permitted free activity after recovery from anesthesia.

All rats fasted for 8 h before euthanasia. The rats in all groups were euthanized by exsanguination *via* the abdominal aorta at observation time points of 2, 4 and 6 weeks under anesthesia. Half of the fractured femurs were harvested and prepared for micro-CT scanning and biomechanical testing at each time point. The others were kept in neutral buffered formalin (10%, pH 7.4) for histological testing. The specimens were decalcified with EDTA (Servicebio, China), dehydrated with different concentrations of alcohol (70% to 100%) and xylene, and finally embedded in paraffin.

### Biochemical analysis of serum biomarkers of oxidative stress levels

Serum marker levels were used to indicate oxidative stress levels in rats. Blood was collected (100 μL) from the abdominal aorta immediately prior to sacrifice. The blood was centrifuged for 16 min at 5,500 RPM and assessed as soon as possible. Serum levels were calculated following the biochemical check method. The effects of HRS with or without 3MA were assessed by analyzing the levels of SOD, MDA and glutathione peroxidase (GSH).

### X-ray photography and micro-CT scan

To observe the process of fracture union, X-rays (Carestream, digital DRX-1 System) were taken to evaluate callus formation on the fracture sites at 2, 4 and 6 weeks after fracture. The parameters in the healing scoring system included gap size, bone in the gap, and mineralized callus, and all of them were assigned scores of 0, 1, or 2 with a composite score ranging from 0 to 6. Furthermore, the middle part of the fractured femoral bones was scanned and evaluated with microcomputed tomography (1176, Skyscan, Bruker, Kontich, Belgium) in our center, and the parameters were set as follows: an image pixel size of 16.62 μm (73 kV, 313 μA) and an acquisition of 360 projections per 180°. All femoral samples were scanned within 1 day after sacrifice.

The region with a width of 7 mm surrounding the osteotomy site proximally and distally (approximately 393 slices) was set as the region of interest (ROI) and selected within the software provided. Values including the percent bone volume/total volume (BV/TV, %), trabecular thickness (Tb. Th, mm), trabecular number (Tb. N, 1/mm), and trabecular separation (Tb. Sp, mm) were recorded and calculated.

### Biomechanical test

The specimens were thawed and immersed in 0.9% saline water (25°C). The fractured femur was bilaterally fixed after removal of the internal fixation (k-wires). The mechanical testing machine (maximal load 225 N, BOSE 3520-AT, USA) was utilized to assess the biomechanical properties in each group with three bending tests (23 mm span, 2 mm/min). The sagittal and coronal widths (the fractured end) were recorded with sliding calipers (Absolute 536, Mitutoyo, Japan). Parameters including the maximum load, stiffness, Young’s modulus and energy were all recorded and calculated. The index values, such as the inner diameter and outer diameter of the fractured section, were also recorded.

### Histopathology and immunohistochemistry

After fixation with 4% paraformaldehyde PBS for 1 day at 4°C, each femur (right) was decalcified for 8-10 weeks in EDTA (pH 7.4, 25°C) and dehydrated in graded alcohol. Then, the paraffin embedded specimens were cut (RM2016, Leica, Shanghai, China) to a sagittal thickness of 4 μm and subjected to hematoxylin and eosin (HE) staining and safranin O/light green staining for histological assessment. Histological evaluation was conducted with light microscopy (Eclipse E100, Nikon, Japan) using an Axiocam ICc3 digital camera (Carl Zeiss). The other slides were used to conduct immunohistochemical staining for LC3 II (1:100, ab48394, Abcam, Shanghai, China), Beclin 1 (1:100, ab62557, Abcam, Shanghai, China), P62 (1:50, ab109012, Abcam, Shanghai, China), Keap1 (1:500, ab226997, Abcam, Shanghai, China), and Nrf2 (1:100, YT3189, Immunoway, Suzhou, China). The intensity of positively stained cells at the femoral fracture region was quantified in five visual fields from each specimen at 200× magnification with integrated optical density utilizing Image-Pro Plus software (Media Cybernetics, USA). The measurement in the sham group was set as the standard value and the measurements for each experimental group were expressed relative to sham (fold of sham).

The undecalcified femur was handled, dehydrated, and embedded in methylmethacrylate to conduct the histomorphometric analysis. A diamond saw (HistoCore AUTOCUT, Leica Instruments, Shanghai) was used to cut the femur longitudinally (~10 μm thick). The ROI was identified covering a total of 7 mm proximal and distal to the fracture site. The decalcified femurs (by Stevenel’s blue and Van Gieson’s picrofuchsin for histomorphometric analysis) were obtained and evaluated (Image-Pro software) to compare the bone histomorphometric parameters, including the bone formation rate per bone surface (BFR/BS), the callus area (Ca. Ar), mineral apposition rate (MAR), and lamellar/Ca.Ar.

### Western blot assay

The right femurs were rapidly removed and placed into liquid nitrogen or stored at -80°C until use. The segments for Western blotting were ground in liquid nitrogen and homogenized on ice in RIPA buffer containing protease inhibitors. Total protein was extracted following the manufacturer’s protocols. Protein concentrations were determined (BCA kit), and the bone samples were then separated by SDS–PAGE and electrotransferred to a polyvinylidene fluoride (PVDF, 0.45 μm) membrane (Roche, Basel, Switzerland; 300 mA for 30 minutes). The membrane was then blocked in TBS buffer containing 5% bovine serum albumin for 30 minutes at 37°C. Then, the membrane was incubated with primary antibodies at 4°C overnight and with HRP-conjugated secondary antibodies for 30 minutes. LC3-II (1:1000, ab48394, Abcam, Shanghai, China), Beclin 1 (1:1000, ab62557, Abcam, Shanghai, China), P62 (1:1000, ab109012, Abcam, Shanghai, China), Keap1 (1:1000, ab226997, Abcam, Shanghai, China), and NRF2 (1:1000, YT3189, Immunoway, Suzhou, China) antibodies were used. β-actin was blotted on the same membranes and served as a control. The bands on the membranes were subsequently imaged using the Odyssey two-color infrared laser imaging system (LI-COR, Lincoln, NE, USA). The relative intensity of each band was normalized to β-actin.

### Statistical analysis

The data were checked for normality and homogeneity of variance and analyzed using SPSS 21. The results are presented as the mean ± standard deviation. Frequencies are used to express the categorical data. The Kolmogorov–Smirnov test was used to examine the normal distribution, and Levene’s test was used for homogeneity of variance. One-way analysis of variance (ANOVA) was applied with the LSD test to determine the statistically significant differences between the sham group and all other ovariectomized groups. The significance level was set at 0.05.

## Results

The rats in the sham group showed a significant increase in oxidative stress levels at 2 and 4 weeks, but showed normal levels as fracture healing or the bone remodeling process was completed at the end of follow-up (6 weeks). The levels of MDA, which reflects the level of oxidative stress, were significantly increased in the OVX group at 2, 4 and 6 weeks, compared with the sham group. After treatment with HRW in the OH group, the level of oxidative stress was significantly decreased. Two weeks into the fracture healing process, MDA was significantly increased in the OHA group (with 3MA treatment) compared with the OH group. At 4 and 6 weeks, however, MDA levels were comparable.

**Figure 1:**
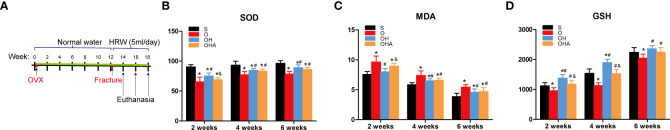
**(A)** A schematic illustration of the osteoporotic fracture treatment model established by OVX with administration of HRW. **(B–D)** Changes in biochemical results in the four groups at different time points after femoral fracture. S, normal control group; O, ovariectomy group; OH, ovariectomy with hydrogen-rich water administration; OHA, ovariectomy with hydrogen-rich water and 3MA administration. Each group contained 6 rats. *p < 0.05 compared to S; ^#^p < 0.05 compared to O; ^&^p < 0.05 compared to OH.

The levels of SOD and GSH-PX were significantly decreased in the OVX group at 2, 4 and 6 weeks, compared with the sham group. After treatment with HRS in the OH group, the levels of SOD and GSH-PX were significantly increased (**Figure 1**). However, when 3MA was administered to the OHA group, SOD and GSH-PX levels were significantly decreased compared with the OH group and increased compared with the OVX group.

### X-ray photography and HE and Safranin O staining

**Figure 2:**
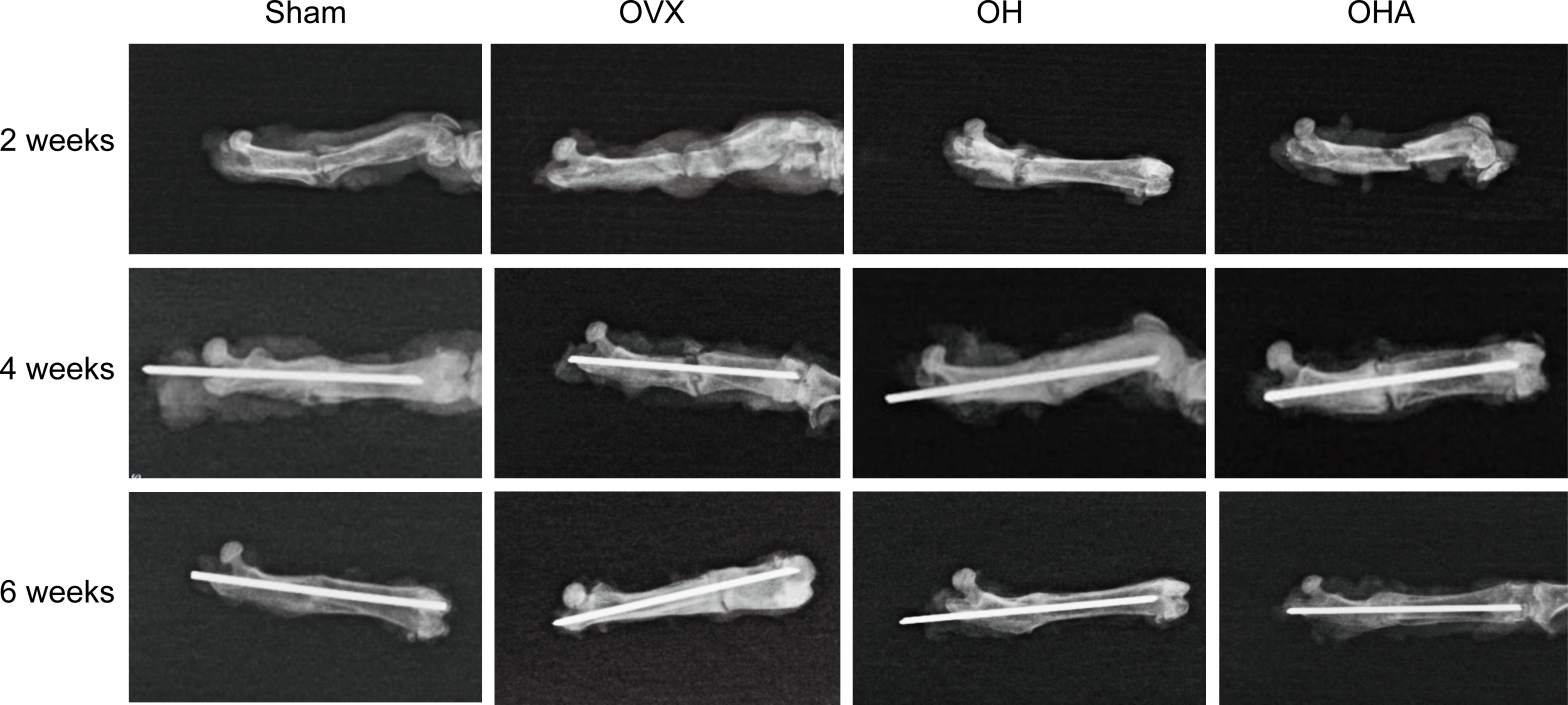
Radiographs in the four groups at different time points after femoral fracture. Radiographs showed that the callus formed in the sham group was characterized by a relatively large portion of high-density bone tissue and fibrocartilage, which was similar to woven bone, and the fracture was partly healed at 2 weeks. At the end of follow-up, a clear reduction in callus size was observed, and the external callus was also gradually remodeled and restored to the original cortical configurations in the OH and OHA groups. Sham, normal control group; OVX, ovariectomy group; OH, ovariectomy with hydrogen-rich water administration; OHA, ovariectomy with hydrogen-rich water and 3MA administration.

**Figure 3:**
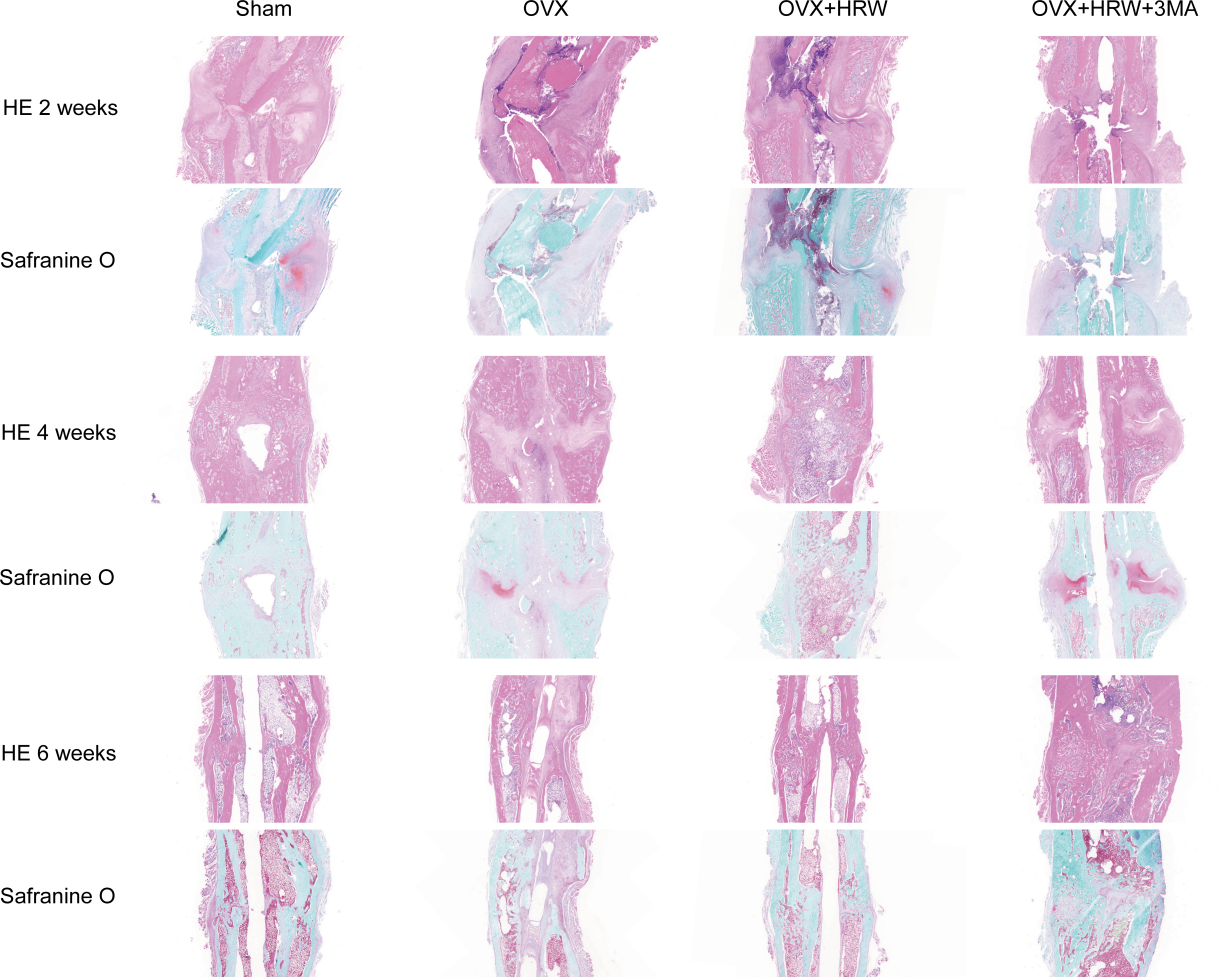
Histological pictures in each group at 2, 4 and 6 weeks after femoral shaft fracture. HE staining, safranin O staining. In the OVX group at 2 weeks, the fracture gap was clearly illustrated with a large amount of low-density bone or inflammatory tissue. There was more high-density mineralized bone formed in the OH group than in the OVX group but less than in the sham group. The OH group exhibited a significant increase in radiographic healing scores and callus compared with other ovariectomized groups. In the OHA group at 2 weeks, the formed callus was low in density and small in size, and the bridging of the fracture ends with newly formed callus was slower than that in the OH group but similar to that in the OVX group. There was a significant increase in fracture healing scores in the OH group compared with the OVX and OHA groups at 4 weeks. The OHA group showed increased healing scores at 4 weeks after treatment with 3MA and then, after 6 weeks, had a similar healing score compared with the OH group. At 6 weeks, a clear reduction in callus size was observed, and the bone remodeling process was almost completed in the sham group. The external callus was also gradually remodeled and restored to the original cortical configurations in the OH and OHA groups, but the callus in the OVX group was still undergoing remodeling, and the woven bone and fracture line still existed. Scale bar = 1000 μm.

Radiographs showed that the callus formed in the sham group was characterized by a relatively large portion of high-density bone tissue and fibrocartilage, which was similar to woven bone, and the fracture was partly healed at 2 weeks. In comparison, in the OVX group at 2 weeks, the fracture gap was clearly observed with a large amount of low-density bone or inflammatory tissue. There was more high-density mineralized bone formed in the OH group than in the OVX group but less than in the sham group (**Figures 2**, **3**). The OH group exhibited a significant increase in radiographic healing scores and callus formation compared with other ovariectomized groups. In the OHA group at 2 weeks, the formed callus was low in density and small in size, and the bridging of the fracture ends with newly formed callus was slower than that in the OH group but similar to that in the OVX group.

**Figure 4:**
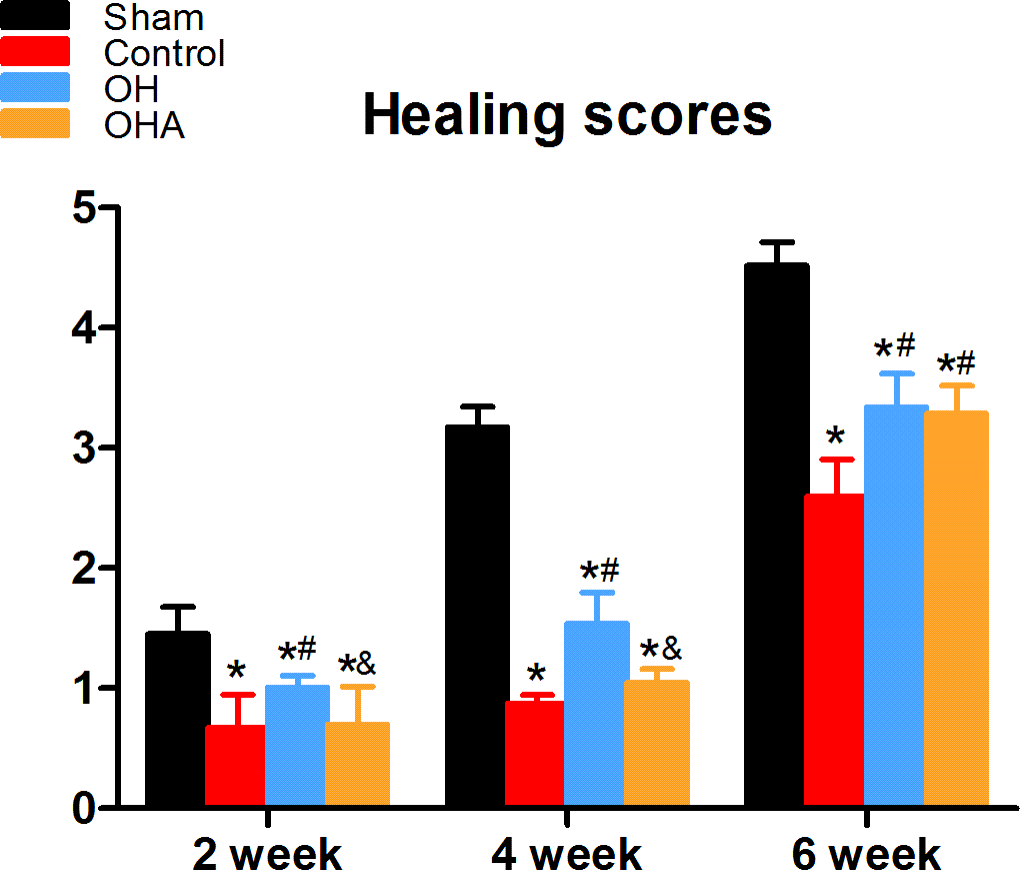
Healing scores in the four groups at different time points (2, 4 and 6 weeks) after femoral fracture. *p < 0.05 compared to S; #p < 0.05 compared to OVX; &p < 0.05compared to OH.

There was a significant increase in fracture healing scores in the OH group compared with the OVX and OHA groups at 4 weeks (P < 0.05). The OHA group showed increased healing scores at 4 weeks after treatment with 3MA and then, after 6 weeks, had a similar healing score as the OH group (**Figures 2**–**4**). At 6 weeks, a clear reduction in callus size was observed, and the bone remodeling process was almost completed in the sham group. The external callus was also gradually remodeled and restored to the original cortical configurations in the OH and OHA groups, but the callus in the OVX group was still undergoing remodeling, and the woven bone and fracture line still existed.

### Micro-CT

**Figure 5:**
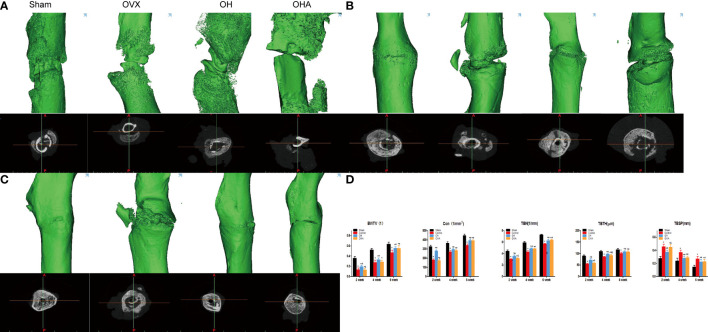
3D reconstruction and axial view of the fractured femur at 2, 4 and 6 weeks. **(A)** 3D reconstructions and axial view of the fracture in the sham, OVX, OH and OHA groups at 2 weeks after fracture. **(B)** 3D reconstructions and axial view of the fracture in the sham, OVX, OH and OHA groups at 4 weeks after fracture. **(C)** 3D reconstructions and axial view of the fracture in the sham, OVX, OH and OHA groups at 6 weeks after fracture. **(D)** 3D histomorphometry of the fractured femur at 2, 4 and 6 weeks. Sham, normal control group; OVX, ovariectomy group; OH, ovariectomy with hydrogen-rich water administration; OHA, ovariectomy with hydrogen-rich water and 3MA administration. *p < 0.05 compared to S; #p < 0.05 compared to OVX; &p < 0.05 compared to OH.

The histomorphometric analysis demonstrated that high-density mineralized bone gradually increased in the sham group at 2 weeks. In comparison, the bridged callus in the OVX group was small and had a low density (woven bone). Consistent with the radiographs, more mineralized bone tissue was observed at the fracture site in the OH group than in the OVX group (**Figure 5**). Furthermore, the OH group showed significantly improved BV/TV, Con, Tb. N, and TBTH compared with the OVX group. However, it was found that the bone healing process was negatively affected in the OHA group at 2 weeks (**Figure 5**). The BV/TV, Tb. Th, Tb. N, and Conn. D were found to be significantly decreased after 3MA was used in the OHA group compared with the OH group (p<0.05), and the parameter was comparable to that in the OVX group.

At 4 and 6 weeks, a remarkable increase in Tb. Th, Tb. N and Conn. D, and a decrease in TBSP in the sham group was observed over time. The OH group exhibited an improvement in bone parameters such as Tb. Th and Tb. N compared with the OVX group. These results verified the positive effect of HRW on bone healing of osteoporotic fractures (**Figure 5**). As the effect of 3MA decreased at 4 and 6 weeks, it was found that the Tb. Th, Tb. N and Conn. D increased gradually in the OHA group. However, no significant differences were found compared with the OH group at 6 weeks, which can be explained by the fact that HRW plays a role in fracture healing and bone remodeling after treatment with 3MA (**Figure 5**).

### Biomechanical testing

The OVX group showed a significant decrease in the ultimate load, energy stiffness and elastic modulus in comparison with the sham group at 2, 4 and 6 weeks. Furthermore, in accordance with our previous bone density analysis, these biomechanical parameters in the OH group were significantly higher than those in the OVX group at 2, 4 and 6 weeks. Furthermore, there were no differences in ultimate load, energy absorption or elastic modulus at 2 or 4 weeks, but there was a significant increase in the OHA group compared with the OVX group at 6 weeks. In terms of stiffness, there was a clear improvement in the OHA group compared with the OVX group at 4 and 6 weeks.

**Figure 6:**
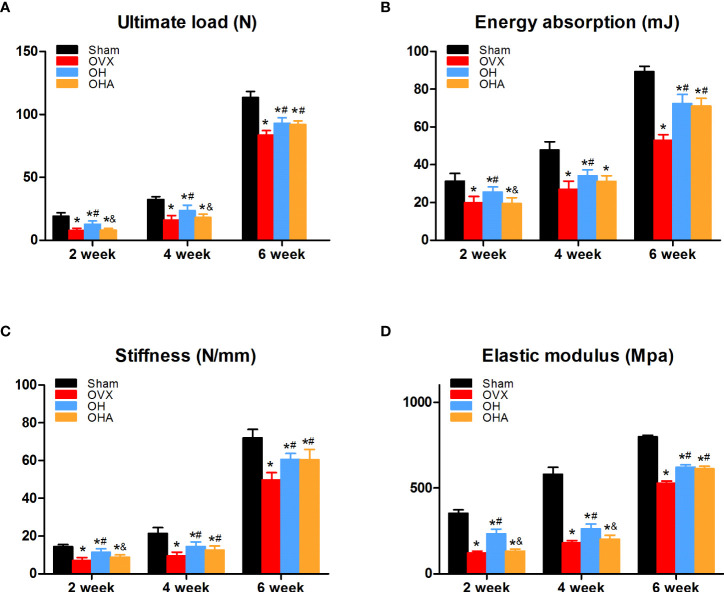
Biomechanical assessment of the femur in the four groups at 2, 4 and 6 weeks. **(A)** Ultimate load changes in different groups. **(B)** Energy absorption changes in different groups. **(C)** Stiffness changes in different groups. **(D)** Elastic modulus in different groups. S, normal control group; OVX, ovariectomy group; OH, ovariectomy with hydrogen-rich water administration; OHA, ovariectomy with hydrogen-rich water and 3MA administration. Each group contained 3 rats. *p < 0.05 compared to S; ^#^p < 0.05 compared to OVX; ^&^p < 0.05 compared to OH.

The OHA group showed a significant decrease in ultimate load, energy absorption, stiffness and elastic modulus in comparison with the OH group at 2 weeks, but no significant differences in energy absorption or stiffness between the OH and OHA groups were observed at 4 weeks. At 6 weeks, no significant differences were observed in these biomechanical parameters in the OHA group compared with those in the OH group (**Figure 6**).

### Bone histomorphometry

Significantly lower Ca. Ar, Lamellar/Ca. Ar, MAR and BFR/BS were observed in the OVX group compared with the sham group at 2, 4 and 6 weeks through quantitative analysis (p < 0.05). Compared with the OVX group, there was a significant increase in bone histomorphometric parameters at 2, 4 and 6 weeks (p < 0.05) in the OH group. Furthermore, no differences in bone histomorphometry were seen in the OHA group compared with the OVX group, but it was significantly decreased compared with the OH group at 2 weeks. After the effect of 3MA disappeared over time, a significant increase in Ca.Ar, Lamellar/Ca. Ar, MAR and BFR/BS were observed in the OHA group compared with the OVX group, and comparable results were found between the OH and OHA groups at 6 weeks (p > 0.05) (**Table 1**).

**Table 1:** Effects of oral administration of HRW and percutaneous administration of 3MA on trabecular bone histomorphometry in rats subjected to ovariectomy combined with femoral fracture.

Bone histomorphometry in fracture callus at 2 weeks post-fracture				
Group	Sham	Control	OH	OHA
Ca.Ar (mm^2^)	9.36±1.70	4.61±1.38^a^	6.44±1.72^a,b^	4.68±1.01^a,c^
Lamellar/Ca.Ar (%)	28.11±5.88	6.9±1.32^a^	18.32±4.08^a,b^	7.59±1.31^a,c^
BFR/BS (μm^3^/μm^2^ per day)	1.37±0.15	0.53±0.15^a^	1.06±0.18^a,b^	0.86±0.14^a,c^
MAR (μm/day)	2.37±0.28	1.26±0.23^a^	1.72±0.12^a,b^	1.48±0. 17^a^
Bone histomorphometry in fracture callus at 4 weeks post-fracture				
Group	Sham	Control	OH	OHA
				
Ca.Ar (mm^2^)	13.69±1.23	7.61±1.02^a^	11.29±1.76^a,b^	7.64±1.55^a,c^
Lamellar/Ca.Ar (%)	47.92±3.52	12.91±2.97^a^	24.46±5.33^a,b^	14.26±2.24^a,c^
BFR/BS (μm3/μm2 per day)	1.11±0.14	0.58±0.11^a^	0.83±0.10^a,b^	0.69±0.13^a,c^
MAR (μm/day)	1.96±0.16	1.24±0.17^a^	1.68±0.19^a,b^	1.45±0.12^a,b,c^
Bone histomorphometry in fracture callus at 6 weeks post-fracture				
Group	Sham	Control	OH	OHA
Ca.Ar (mm^2^)	11.7±1.57	7.13±1.34^a^	9.26±1.45^a,b^	9.15±1.56^a,b^
Lamellar/Ca.Ar (%)	91.46±2.18	76.67±3.85^a^	85.85±3.44^a,b^	85.03±5.55^a,b^
BFR/BS (μm^3^/μm^2^ per day)	1.11±0.14	0.48±0.10^a^	0.65±0.13^a,b^	0.63±0.13^a,b^
MAR (μm/day)	1.74±0.15	1.13±0.13^a^	1.45±0.11^a,b^	1.43±0. 17^a,b^

Values represent the mean ± SD. Each group was formed by 6 rats. ^a^p<0.05 compared to Sham. ^b^p<0.05 compared to Control. ^c^p<0.05 compared to OH.

### Immunohistochemistry

To investigate how HRW affects the level of autophagy and antioxidative stress response in the osteoporotic fracture healing process, LC3-II, Beclin 1, P62, Keap1 and Nrf2 expression levels were examined.

**Figure 7:**
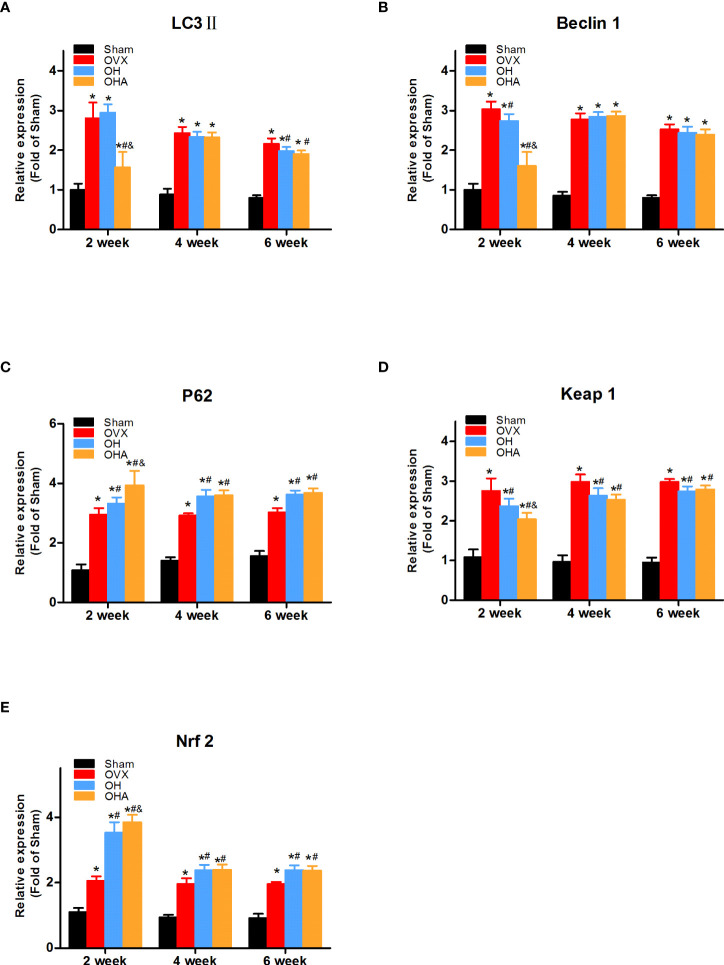
Quantitative comparison of immunohistochemistry results in four groups at 2, 4 and 6 weeks. **(A)** LC3II expression in different groups. **(B)** Beclin 1 expression in different groups. **(C)** P62 expression in different groups. **(D)** Keap1 expression in different groups. **(E)** Nrf2 expression in different groups. S, normal control group; OVX, ovariectomy group; OH, ovariectomy with hydrogen-rich water administration; OHA, ovariectomy with hydrogen-rich water and 3MA administration. Each group contained 3 rats. *p < 0.05 compared to S; ^#^p < 0.05 compared to OVX; ^&^p < 0.05 compared to OH.

**Figure 8:**
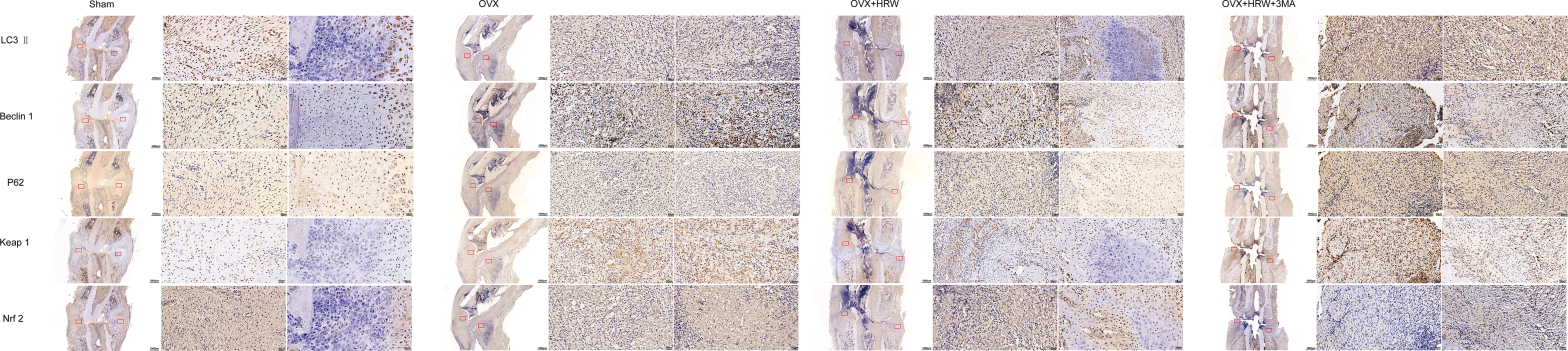
Immunohistochemistry results for autophagy and Keap1-Nrf2 signaling in the four groups at 2 weeks. The expression levels of LC3-II, Beclin 1, P62 showed that autophagy and the antioxidative stress system in the OVX group were further activated during fracture healing to improve the fitness of chondrocytes and osteoblasts compared with the sham group at 2 weeks. At 2 weeks, the antioxidative stress system Keap1-Nrf2 was partly activated in the OHA group. Scale bar = 1000 μm and 50 μm.

The expression levels of LC3-II, Beclin 1, P62, Keap1 and Nrf2 showed that autophagy and the antioxidative stress system in the OVX group were further activated during fracture healing to improve the fitness of chondrocytes and osteoblasts compared with the sham group at 2 weeks. In the OH group, there was a significant decrease in Beclin 1 and an increase in P62, and the expression of Keap1 in the cytoplasm was also significantly decreased compared with that in the OVX group (P < 0.05). In addition, Nrf2 was more highly expressed in the nucleus, which means that the antioxidative stress system was activated in those cells so that the function of bone repair-relevant cells could be maintained in a relatively stable state to achieve rapid repair of fractures. In the OHA group, the expression of LC3-II and Beclin 1 was significantly decreased compared with that in the sham, OVX and OH groups, while P62 was sharply increased at 2 weeks. At 2 weeks, the antioxidative stress system Keap1-Nrf2 was partly activated in the OHA group (**Figures 7**, **8**).

**Figure 9:**
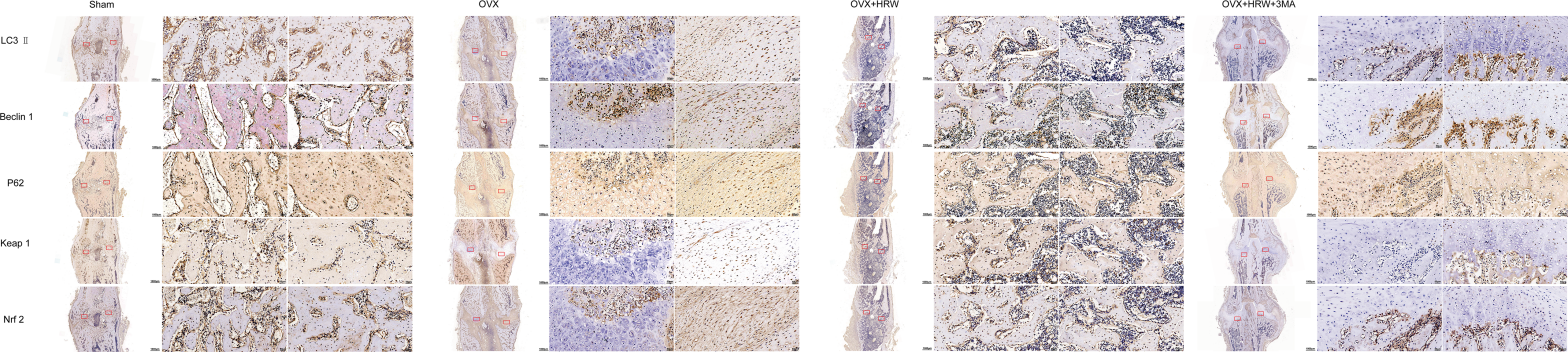
Immunohistochemistry results for autophagy and Keap1-Nrf2 signaling in the four groups at 4 weeks. At 4 weeks in the OVX group, although autophagy was clearly activated, it was still not enough to achieve a balance between oxidative stress and autophagy. A significant increase in the levels of P62 and Nrf2 was observed in the OH group compared with the OVX group. With 3MA treatment in the OHA group, the expression levels of LC3-II and Beclin 1 gradually increased, and the autophagy level was normalized. The level of Nrf2 in the OHA group was also significantly increased compared with that in the OVX group and was comparable to that in the OH group due to the application of HRW. Scale bar = 1000 μm and 50 μm.

At 4 weeks in the OVX group, although autophagy was clearly activated, it was still not enough to achieve a balance between oxidative stress and autophagy. Therefore, the overall environment was still unfavorable for chondrocyte or osteoblast function. Furthermore, a significant increase in the levels of P62 and Nrf2 was observed in the OH group compared with the OVX group (P <0.05). With 3MA treatment in the OHA group, the expression levels of LC3-II and Beclin 1 gradually increased, and the autophagy level was normalized. The level of Nrf2 in the OHA group was also significantly increased compared with that in the OVX group (P <0.05) and was comparable to that in the OH group due to the application of HRW (**Figures 7**, **9**).

**Figure 10:**
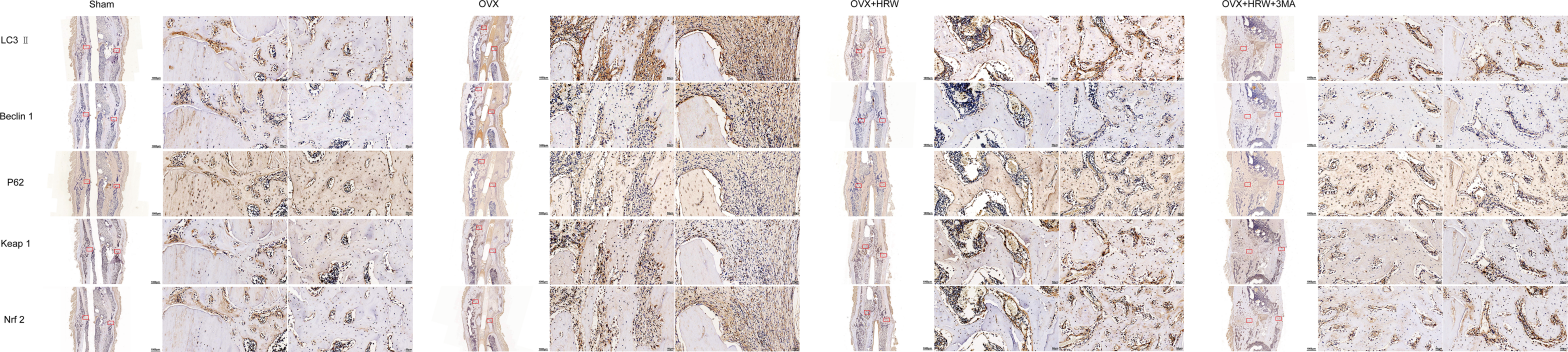
Immunohistochemistry results for autophagy and Keap1-Nrf2 signaling in the four groups at 6 weeks. At 6 weeks, the autophagy level in the sham group was decreased to a stable state. In the OVX group, high levels of oxidative stress and autophagy were observed, part of the bone tissue was undergoing bone remodeling, and dead bone was also observed at the fracture site. A significant increase in the level of P62 was observed in the OH and OHA groups compared with the OVX group. Furthermore, the expression of Nrf2 in the OH and OHA groups tended to be normal but still high compared with that in the sham group, but compared with that in the OVX group, the level of autophagy was low, and the antioxidative stress response was strong. Scale bar = 1000 μm and 50 μm.

At 6 weeks, the autophagy level in the sham group was decreased to a stable state, at which point the process of callus remodeling was almost complete. In the OVX group, high levels of oxidative stress and autophagy were observed, part of the bone tissue was undergoing bone remodeling, and dead bone was also observed at the fracture site. In the OH and OHA groups, more lamellar calli were formed than in the OVX group, and the autophagy level tended to be normal and lower than that in the OVX group (**Figures 7**, **10**). A significant increase in the level of P62 was observed in the OH and OHA groups compared with the OVX group. Furthermore, the expression of Nrf2 in the OH and OHA groups tended to be normal but still high compared with that in the sham group, but compared with that in the OVX group, the level of autophagy was low (P <0.05), and the antioxidative stress response was strong.

### Western blot analysis

The expression levels of LC3-II, Beclin 1, and P62 in the fractured region were measured to illustrate the level of autophagy. Significantly increased levels of LC3-II, Beclin 1 and P62 expression were observed in the OVX group compared with the sham group. P62 expression was slightly increased in the OH groups compared with the OVX group, meaning that autophagy was partly suppressed at 2 weeks in the OH group. Furthermore, after the administration of HRW, Keap1 expression was significantly decreased, and Nrf2 expression was increased in the OH group at 2 weeks, meaning that the Keap1-Nrf2 pathway antioxidative system was more highly activated in the OH group than in the OVX group. If autophagy was significantly suppressed with 3MA, the antioxidative system was also activated in the OHA group, but bone formation was not improved, which may be explained by the relatively normal level of autophagy needed in the fracture healing process.

At 4 weeks, autophagy was still activated in the OVX, OH and OHA groups, but after stopping 3MA treatment in the OHA group, the expression levels of LC3-II, Beclin 1 and P62 were comparable to those in the OH group, and the autophagy level was still lower than that in the OVX group. Furthermore, the expression level of Keap1 was lower in the OH and OHA groups than in the OVX group, meaning that the Keap1-Nrf2 signaling pathway was still activated. These results were verified by the increased expression of Nrf2 at 4 weeks.

**Figure 11:**
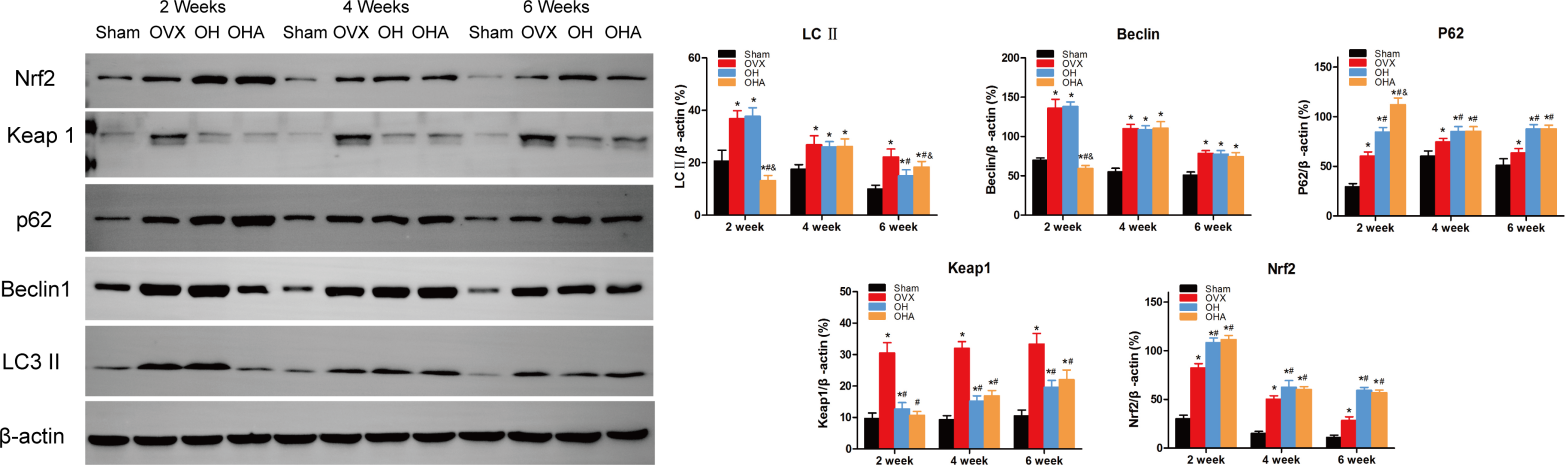
Western blot analysis in four groups at 2, 4 and 6 week. Representative Western blot bands and relative expression levels for LC3-II, Beclin1, P62, Keap1 and Nrf2 at 2, 4 and 6 weeks after fracture. *p < 0.05 compared to S; ^#^p < 0.05 compared to OVX; ^&^p < 0.05 compared to OH.

At 6 weeks, the bone remodeling process was observed in most specimens of the sham group, and the expression of LC3-II, Beclin 1, P62 (autography level), Keap1, and Nrf2 (Keap1-Nrf2 signaling system) was normal compared with that of the OVX group, in which woven bone was still observed. In the OH and OHA groups, autophagy was sustained at a relatively stable lower level compared with that in the OVX group, and antioxidative stress pathway activation was still high compared with that in the OVX group (**Figure 11**).

## Discussion

Autophagy maintains intracellular homeostasis by degrading and circulating metabolites, providing nutrients, and eliminating many cytotoxic substances (i.e., damaged proteins). It is a crucial mediator and is expanded in the pathological response to oxidative stress (7). The accumulation of oxidative stress causes cellular constituents, such as proteins, DNA and lipids, to be oxidized and damaged, which activates the autophagic process (8). Similar to oxidative stress, stable autophagy also plays an essential role in maintaining an appropriate balance of osteoclasts and osteoblasts during bone remodeling (9). HRW, as an antioxidant molecule, selectively reduces the levels of strong oxidants, but its effect on the osteoporotic fracture healing process (mainly affected by osteoblast cell activities) has not been comprehensively elucidated. Radiographic, biomechanical, and histopathologic results illustrate that autophagy is an essential mechanism and that basal levels of autophagy are necessary in the osteoporotic fracture healing process. Furthermore, HRW treatment slightly suppresses autophagy and activates the Keap1-Nrf2 signaling pathway by maintaining the P62-Keap1-Nrf2 interaction and improves the osteoporotic fracture healing process.

Osteoporosis is characterized by decreased bone density and bone mass and the destruction of bone microstructure, and it is due to genetic, hormonal and nutritional factors, resulting in increased bone fragility or fracture. Age-related bone loss is associated with increased oxidative stress, and in women, this is aggravated by decreased estrogen during menopause. In our previous research, HRW was proven to preserve bone volume and decrease fracture risks in streptozotocin-induced diabetic rats. To further study the osteoporotic bone or fracture healing process, ovariectomized rats were used to illustrate the potential mechanism of HRW and to try to illustrate how to reverse the shift in the balance of bone formation and absorption toward formation (osteoblasts) to promote fracture healing. In the early stage of fracture healing, the level of oxidative stress rises, and the body is in a state of high oxidative stress. As the fracture healing process is gradually completed, oxidative stress gradually decreases and reaches a balance in the normal fracture group. The oxidative stress level was the lowest in the sham group, and significantly higher in the OVX group. After the application of HRW, the higher oxidative stress level was significantly alleviated. As an antioxidant, HRW has an anti-inflammatory effect and selectively reacts with hydroxyl radicals, which are the most cytotoxic ROS in tissues. Furthermore, as the major inducible system to maintain redox homeostasis, Nrf2 signaling, which senses endogenous or exogenous oxidative stress and is always maintained at low levels by its inhibitor Keap1, was found to be activated in the OVX group, in which the balance between ROS generation and clearance by cellular antioxidant systems was disrupted. Furthermore, Keap1-Nrf2 signaling was further activated in the OH group compared with the OVX group, which means that the antioxidant ability was further improved. In accordance with our results, earlier studies also showed that female Nrf2 KO mice exhibited a deficit in postnatal bone acquisition and increased bone loss (10). Nrf2 activity also promotes osteocyte-specific expression of Dmp1 in IDG-SW3 cells, osteoblasts, and osteocytes in models with Nfe2l2 deficiency (11). Therefore, it was believed that an increase in Nrf2 expression was beneficial to bone homeostasis and the fracture healing process.

In addition to activating Keap1-Nrf2 signaling, autophagy also antagonizes cellular stress by upregulating a battery of antioxidant and cellular defense genes and is responsible for the clearance of damaged organelles, long-lived proteins, or misfolded proteins. This degradation process acts to preserve cellular homeostasis and to defend against oxidative or proteotoxic stress (12). It was reported that decreased autophagy in female osteoblasts is associated with increased oxidative stress and may play a role in the pathophysiology of osteoporosis (13). In osteoblast-specific autophagy-deficient mice, researchers found that autophagy deficiency aggravated bone loss associated with aging and estrogen deprivation. However, as a commonly encountered stress condition, autophagy status during the fracture healing process has not received enough researcher attention. Autophagy alleviates damage, but levels of autophagy in postmenopausal patients are insufficient to alter the negative balance of oxidative stress, and certain extra interventions are needed to alleviate this increasing imbalance. Our research illustrated that the level of autophagy in the sham group was increased at the beginning of callus formation and then returned to normal after bone remodeling was completed. Furthermore, the increased oxidative stress in ovariectomized rats was reported to induce autophagy. In accordance with other studies, the level of autophagy was indeed found to be increased during the fracture healing process in the OVX group compared with that in the sham group. However, after the application of HRW, autophagy was slightly suppressed, Keap1-Nrf2 signaling was activated, and the fracture healing process was improved in the OH group compared with the OVX group. It should be noted that a relatively high level of autophagy was required for fracture healing, and excessive inhibition of autophagy was not conducive to fracture healing in the OHA group. The results of the OH and OHA groups seem to be contradictory, but the slightly suppressed autophagy in the OH group may be the result of a combined effect. Researchers have reported that antioxidant molecules can moderately or completely suppress autophagic flux, which is in agreement with our study, but the potential mechanism is complex (14). One potential reason is that autophagic flux was blocked due to increased levels of LC3 II and P62 in the OH group compared with the OVX group. Another reason might be that with HRW application, the number of osteoblasts, osteoclasts or even osteocytes with normal function was increased, so the action needed to restore metabolic homeostasis through the degradation of macromolecules was decreased. To explain why slightly suppressed autophagy activates the expression of Nrf2, the potential relationship between the Keap1-Nrf2 system and autophagy status also needs to be examined further in the process of fracture healing.

The Keap1-Nrf2 signaling pathway and autophagy were shown to intersect through the direct interaction between P62 and Keap1 (15–17). The Keap1-interacting region domain of p62 resembles the Keap1-interacting ETGE motif of Nrf2, which clearly accounts for the interaction between p62 and Keap1 (15, 17). It allows P62 to sequester Keap1 and move into autophagosomes, damaging the ubiquitylation of Nrf2, followed by Nrf2 translocation into the nucleus and activation of the Nrf2 signaling pathway, which is called P62-dependent Nrf2 activation (noncanonical pathway). In addition to the sequestration of Keap1 into autophagosomes, Keap1 is also considered a P62-regulated substrate in autophagy-mediated degradation. Furthermore, P62 has been observed to play a key role in controlling Keap1 turnover, as researchers found that overexpression of P62 significantly decreased the half-life of Keap1, whereas deficiency of P62 increased it (18). It was illustrated that in wild-type mice, the level of Keap1 was much lower than that in Atg7-deficient or P62-deficient mice (19). These results indicated that P62 mediates Keap1 turnover, sequesters Keap1 and contributes to the noncanonical activation of Nrf2. On the other hand, evidence supporting the concept that the Keap1-Nrf2 pathway also regulates the autophagy system is emerging (14, 20). Nrf2 can induce P62 gene expression in response to oxidative stress, after which the protein further activates Nrf2, forming a positive feedback loop (15, 17, 21). Therefore, these proteins controlling responses to oxidative stress and autophagy show the functional intersection between the Keap1-Nrf2 pathway and the autophagy-lysosomal pathway. In accordance with those studies, our results show that P62 was increased in the OH group compared with the OVX group. The increased P62 level in the OH group was beneficial to the activation of Nrf2 through the noncanonical pathway. Along with the increased level of P62, Nrf2 was also activated in the OHA group. However, the main difference in the OHA group compared with the OH group was that the autophagy level was significantly decreased, which was essential to the normal fracture healing process. Therefore, the increased Nrf2 expression caused by HRW, not 3MA, had a positive effect on the fracture healing process. However, Ni et al. reported that when autophagy was ablated due to deletion of Atg5, Beclin 1 and P62-Keap1 aggregated and accumulated in the cytosol, resulting in prolonged Nrf2 activation, which protected against tissue injury (22). It should be noted that the temporary persistent Nrf2 activation in the fracture healing process was not caused by genetic mutations, so it was different than the gene deletion model or cancer, in which it has been recognized to be pathogenic, illustrating the potential negative effect of Nrf2. Therefore, it can be concluded that the P62-Keap1-Nrf2 interaction in autophagy regulation and redox signaling was maintained, and Nrf2 was activated to coordinate the intracellular oxidant/antioxidant status after the application of HRW in the osteoporotic fracture healing process.

One limitation of this study is that it did not include knockout mice to verify the results, but the treatment of osteoporotic fractures has always been difficult, so the research was still beneficial for the formulation of new treatment strategies. Furthermore, bone turnover, which is considered important for illustrating bone remodeling, as evidenced by cross-linked C-terminal telopeptide of type I collagen, procollagen type 1 N-terminal propeptide osteoprotegerin, and runt-related transcription factor 2 protein expression, was not evaluated here, but the macroscopic indicators of bone measurements used in this article can still suggest changes in bone metabolism (23–25).

## Conclusion

After identifying the interaction between the Keap1-Nrf2 axis and autophagy, it was concluded that HRW treatment activated the Keap1-Nrf2 pathway to antagonize cellular stress by suppressing autophagy levels, especially at the early stage of the fracture healing process, and was beneficial to osteoporotic fracture healing in rats.

## Data availability statement

The original contributions presented in the study are included in the article/supplementary material. Further inquiries can be directed to the corresponding authors.

## Ethics statement

The animal study was reviewed and approved by the ethics committee of the Third Hospital of Hebei Medical University.

## Author contributions

Conceptualization: YZ. Data curation: ST, ZW. Formal analysis: WD. Investigation: YW. Methodology: JG. Project administration: JG. Resources: YZ, WD. Software: JG. Supervision: ZH, YZ. Validation: ZH, YZ. Visualization: ZH. Writing – original draft: JG. Writing – review & editing: JG. All authors contributed to the article and approved the submitted version.

## Funding

The research was supported by the research was supported by the Science and Technology Project of Hebei Education Department (SLRC2019046), Government-funded Clinical Medicine Outstanding Talent Training Project (2019), National Natural Science Foundation of China (82072523, 82002281) and Natural Science Foundation of Hebei (H2020206193, H2021206054), the Main Medical Scientific Research of Hebei (20210543), and China Postdoctoral Fund (2021 M701785), and the 14th Five-Year Clinical Medicine Innovation Research Team (2022). The funders had no role in study design, data collection and analysis, decision to publish, or preparation of the manuscript.

## Conflict of interest

The authors declare that the research was conducted in the absence of any commercial or financial relationships that could be construed as a potential conflict of interest.

## Publisher’s note

All claims expressed in this article are solely those of the authors and do not necessarily represent those of their affiliated organizations, or those of the publisher, the editors and the reviewers. Any product that may be evaluated in this article, or claim that may be made by its manufacturer, is not guaranteed or endorsed by the publisher.
